# Qigui didang decoction alleviates renal injury in a diabetic kidney disease model with metabolic memory features: association with ferroptosis and the SIRT1/Nrf2 pathway

**DOI:** 10.1080/13880209.2026.2648168

**Published:** 2026-03-24

**Authors:** Yusen Wu, Lifei Fan, Hongyi You, Yuhui Lu, Min Lin

**Affiliations:** Fujian University of Traditional Chinese Medicine, Fuzhou, People’s Republic of China

**Keywords:** Qigui Didang Decoction, SIRT1/Nrf2 signaling pathway, ferroptosis, diabetic kidney disease, metabolic memory

## Abstract

**Objective:**

This study aims to evaluate the renoprotective effects of QGDD in a DKD model exhibiting metabolic memory features and to explore its potential mechanism involving the regulation of ferroptosis *via* the SIRT1/Nrf2 signaling pathway.

**Methods:**

A DKD rat model was induced using streptozotocin (STZ). The rats were assigned to the Blank Control Group (C), Model Group (M), Metformin group (Met), and low-, medium-, and high-dose QGDD groups (QGDD-L/M/H). Intervention effects were assessed by monitoring body weight, fasting blood glucose, renal function markers (24-UTP, BUN, Scr, Cys-C, β2-MG), renal histopathology (HE/Masson staining), oxidative stress markers (Fe^2+^, MDA, GSH, SOD), cell death indicators (TUNEL, ROS), and expression of genes and proteins associated with the SIRT1/Nrf2 pathway (RT-qPCR, Western blot).

**Results:**

All QGDD dose groups decreased 24-hour urinary protein excretion and serum levels of BUN, Scr, Cys-C, and β2-MG. The medium-dose QGDD group notably reduced renal Fe^2+^ and MDA levels, increased GSH and SOD activity, and inhibited ROS accumulation and cellular apoptosis. QGDD activated the SIRT1/Nrf2 pathway, significantly upregulating the mRNA and protein expression of Nrf2, HO-1, and GPX4, while suppressing the accumulation of AGEs and Ferritin.

**Conclusion:**

QGDD mitigated the persistent elevation of AGEs, a hallmark of metabolic memory in DKD by activating the SIRT1/Nrf2 signaling pathway to inhibit ferroptosis-associated lipid peroxidation and oxidative stress. Its multi-target synergistic effects provide a solid experimental foundation for the use of Chinese herbal formulations in treating DKD. The medium-dose group demonstrated optimal therapeutic efficacy, emphasizing the significance of dose optimization.

## Introduction

Diabetic kidney disease (DKD) is a chronic renal condition resulting from diabetes and represents one of the most severe microvascular complications of the disease. Clinically, it is characterized by persistent proteinuria and a progressive decline in glomerular filtration rate. With the increasing prevalence of diabetes, it has become the leading cause of chronic kidney disease (CKD). The clinical management of DKD presents considerable challenges. While current first-line therapies can slow the progression of DKD, they do not halt or reverse its course, with progression to end-stage renal disease remaining common (Alicic et al. 2017). This progression imposes significant economic burdens on patients and severely affects survival rates. hyperglycemia is such a significant clinical problem that even subclinical fluctuations in blood glucose, which lead to increased glycated hemoglobin levels but do not qualify for a diagnosis of diabetes, contribute to increased cardiovascular risk and translate into the presence of subclinical cardiovascular dysfunction (Jakubiak et al. 2024; 2025). A key challenge in DKD management is the ‘metabolic memory’ phenomenon, wherein prior periods of hyperglycemia can induce persistent epigenetic and oxidative stress alterations, leading to continued organ damage even after glycemic control is achieved. This perpetuates the progression of renal injury, highlighting the need for therapeutic strategies that target these sustained pathological processes beyond glucose-lowering alone. The pathogenesis of DKD is multifaceted, involving oxidative stress, inflammation, and cell death. Among these, ferroptosis—a form of programmed cell death driven by iron-dependent lipid peroxidation—has recently been linked to the progression of DKD (Zhang et al. 2023; Liu et al. 2024; Yuan et al. 2025). Recent studies have identified the metabolic memory effect induced by chronic hyperglycemia, wherein persistent oxidative stress and epigenetic changes in high-glucose environments continue to damage organs, as a critical factor in the progression of DKD.(Villeneuve and Natarajan 2010; Keating and El-Osta 2013; Kushwaha et al. 2020; Li et al. 2022). This phenomenon highlights the limitations of traditional glucose-centric treatment strategies and underscores the need to explore new therapeutic targets by epigenetically regulating metabolic memory.

Ferroptosis is intricately linked to metabolic memory in DKD (He et al. 2022). SIRT1 stabilizes Nrf2, promoting its nuclear translocation and subsequent activation of antioxidant genes, including GPX4(Chen et al. 2025; Zhang et al. 2025). The SIRT1/Nrf2 redox regulatory axis boosts cellular antioxidant capacity by activating Nrf2, modulating the expression of downstream antioxidant genes like HO-1 and GPX4, and suppressing lipid peroxidation (Sun et al. 2016), thus inhibiting ferroptosis. There exists a bidirectional regulatory relationship between the SIRT1/Nrf2 pathway and metabolic memory. High glucose levels inhibit SIRT1 activity, leading to Nrf2 acetylation and accelerating its ubiquitin-mediated degradation, which exacerbates oxidative stress (Lu et al. 2023). Conversely, activating SIRT1 can reverse this process. For example, curcumin significantly reduces ROS levels in HK-2 cells *via* SIRT1-mediated deacetylation of Nrf2 (Ji et al. 2023).

Qigui Didang Decoction (QGDD), a Chinese herbal formula derived from the classical prescriptions Didang Decoction and Danggui Buxue Decoction, consists of Astragalus membranaceus, Angelica sinensis, Rhubarb, Leech, Gadfly, and Peach Kernel. Based on traditional Chinese medicine principles, this formula synergistically combines dredging and tonifying actions to remove stagnant blood and promote the production of new blood. Previous network pharmacology studies conducted by our research team have demonstrated that QGDD exhibits multi-component, multi-target, and multi-pathway properties. It shows promise in ameliorating DKD by regulating ‘metabolic memory’ through modulation of the AGE-RAGE signaling axis and the PI3K/Akt pathway (Fan et al. 2024; Guo et al. 2024). Additionally, derivatives of Didang Decoction (e.g., Zhijun Tangshen Decoction) improve mitochondrial function by activating the HIF-1 pathway, thereby inhibiting both ferroptosis and apoptosis (Tong et al. 2024). Active components in QGDD, such as Astragaloside IV and Angelica sinensis polysaccharides, significantly upregulate the expression of SIRT1 and Nrf2. For instance, early intervention with Didang Decoction in diabetic rat models enhances mitochondrial energy metabolism through the AMPK/SIRT1 pathway, delaying the onset of macrovascular complications (Ren et al. 2017). Another study confirmed that serum from Didang Decoction inhibits ferroptosis by modulating the Nrf2/HO-1 axis, reducing lipid peroxidation in renal tubular epithelial cells (Dong et al. 2025).

Based on the above evidence, we hypothesize that QGDD may ameliorate metabolic memory in DKD by activating the SIRT1/Nrf2 signaling pathway and subsequently inhibiting ferroptosis. Therefore, this study aimed not only to evaluate the therapeutic effects of QGDD on renal function, pathological injury, and glucolipid metabolism in long-term Streptozotocin (STZ) -induced DKD rats, but also, more importantly, to elucidate whether its renoprotective mechanism involves the modulation of the SIRT1/Nrf2 pathway and key downstream molecules related to oxidative stress and ferroptosis (e.g., GPX4, HO-1). This work seeks to provide experimental evidence and a theoretical foundation for the clinical application of QGDD. Therefore, this study aims to utilize a long-term STZ-induced diabetic kidney disease (DKD) rat model, which recapitulates key features of metabolic memory (e.g., sustained oxidative stress and functional decline), and explored its potential association with the modulation of ferroptosis and the SIRT1/Nrf2 axis, Based on evaluating the ameliorative effects of QGDD on renal function, pathological injury, and glucose-lipid metabolism, the study focuses on investigating whether its renal protective effects are mediated through the regulation of the SIRT1/Nrf2 pathway and its downstream key molecules involved in oxidative stress and ferroptosis (such as GPX4 and HO-1). This research provides experimental evidence for elucidating its mechanism of action and supporting its clinical translation.

## Materials and methods

### Animals

From January 2024 to July 2024, Seventy-two Male SPF SD rats with weight around 200 g were used in this study, all sourced from the Laboratory Animal Center of Fujian University of Traditional Chinese Medicine, with production license number SYXK (Min) 2019-0007. The housing conditions were maintained at an ambient temperature of approximately 22 °C and a humidity range of 36% to 46%. Rats had ad libitum access to food and water, with a 12-hour light/dark cycle.

### Drugs and reagents

Composition of QGDD: The formula includes *Huangqi* 30 g, *Danggui* 6 g, *Dahuang* 10 g, *Shuizhi* 3 g, *Mengchong* 1.5 g, and *Taoren* 5 g. Specifically, *Huangqi* is derived from the root of *Astragalus membranaceus var. mongholicus (Bunge) P. K. Hsiao (Fabaceae)*; *Danggui* is the dried root of *Angelica sinensis (Oliv.) Diels (Apiaceae)*; *Dahuang* is the rhizome of *Rheum palmatum L. (Polygonaceae)*; *Shuizhi* refers to the dried whole body of *Whitmania pigra Whitman (Hirudinidae)*; *Mengchong* represents the dried female whole insect of *Tabanus bivittatus* or related species *(Tabanidae)*; *Taoren* is the dried mature seed of *Prunus persica (L.) Batsch (Rosaceae).* All these herbal decoction pieces were purchased from Jiangsu Guoyao Group.

The decoction was prepared by Jiangsu Guoyao Group. Prior to preparation, Prunus persica seeds were crushed. The extraction process involved boiling the herbs at high heat for 30 min to obtain 200 mL of decoction, followed by a secondary extraction with additional water at low heat for another 30 min to yield a further 200 mL. The combined extracts were then concentrated to a final volume of 100 mL and stored at 4 °C.

Reagents and Kits: Metformin (Solarbio, Cat# D9351); STZ (Solarbio, Cat# S8050); Hematoxylin (Servicebio, Cat# G1004); Eosin (Solarbio, Cat# G1100); Masson’s trichrome staining kit (Servicebio, Cat# G1006); Prussian blue staining kit (Solarbio, Cat# G1422); Modified Oil Red O staining kit (Solarbio, Cat# G1263); Assay kits for reduced glutathione (GSH, Nanjing Jiancheng Bioengineering Institute, Cat# A006-1-1), total cholesterol (TCH/T-CHO, Cat# A111-1-1), triglycerides (TG, Cat# A110-1-1), total superoxide dismutase (T-SOD, Cat# A001-3-2), malondialdehyde (MDA, Cat# A003-1-2), blood urea nitrogen (BUN, Cat# C013-2-1), creatinine (Cr, Cat# C011-2-1), high-density lipoprotein cholesterol (HDL-C, Cat# A112-1-1), low-density lipoprotein cholesterol (LDL-C, Cat# A113-1-1), urinary protein quantification (Cat# F006-1-1), and cystatin C (Cys-C, Cat# C035-2-1); Ferrous ion detection kit (Solarbio, Cat# BC5415); Rat cystatin C (Cys-C) ELISA kit and rat β2-microglobulin (β2-MG) ELISA kit (Jiangsu Meimian Industrial Co., Cat# MM-0438R1 and MM-21246R1, respectively); TUNEL apoptosis detection kit (Servicebio, Cat# G1502); DAPI (Beyotime, Cat# C1006); Dihydroethidium (MCE, Cat# PD-MY003); BCA protein assay kit (Boster Biological, Cat# AR0146); Primary antibodies: Nrf2 (Proteintech, Cat# 80593-1-RR), HO-1 (Cat# 10701-1-AP), GPX4 (Cat# 67763-1-Ig), GAPDH (Cat# 60004-1-Ig); ECL chemiluminescence kit (Wuhan Sanying Biotechnology, Cat# PK10003); Secondary antibodies: HRP-conjugated (Proteintech, Cat# SA00001-1), fluorescein-labeled goat anti-rabbit IgG (Cat# SA00001-2), Urethane (SCR, Cat# 20210521).

### Experimental methods

#### Liquid Chromatography-Mass spectrometry analysis

##### Sample preparation

Samples were thawed slowly on ice. A 50 mg aliquot of each sample was placed into a 2 mL centrifuge tube, followed by the addition of 800 μL of 80% methanol. The mixture was homogenized at a frequency of 65 Hz for 90 s and vortexed thoroughly to ensure complete mixing. Ultrasonication was then performed at 4 °C for 30 min. Subsequently, the samples were stored in a −20 °C freezer for 1h, followed by high-speed centrifugation at 4 °C and 12,000 rpm for 15 min. After centrifugation, 200 μL of the supernatant was aspirated using a micropipette, mixed with 5 μL of internal standard (0.14 mg/mL dichlorophenylalanine), and transferred into an injection vial after thorough mixing.

##### Instrumental analysis

The analysis was performed using an LC-MS system and a chromatographic column. The chromatographic separation conditions were as follows: column temperature, 40 °C; flow rate, 0.3 mL/min; mobile phase A, water containing 0.05% formic acid; mobile phase B, acetonitrile; injection volume, 5 μL; and autosampler temperature, 4 °C.

##### Mass spectrometry parameters

Positive Ion Mode: Heater temperature, 300 °C; sheath gas flow rate, 45 arb; auxiliary gas flow rate, 15 arb; sweep gas flow rate, 1 arb; electrospray ionization (ESI) voltage, 3.0 kV; capillary temperature, 350 °C; S-Lens RF level, 30%.

Negative Ion Mode: Heater temperature, 300 °C; sheath gas flow rate, 45 arb; auxiliary gas flow rate, 15 arb; sweep gas flow rate, 1 arb; ESI voltage, 3.2 kV; capillary temperature, 350 °C; S-Lens RF level, 60%.

##### Scan mode and resolution

Data were acquired using full MS scans (mass-to-charge ratio, m/z 70–1050) with a mass resolution of 70,000, followed by data-dependent MS^2^ scans (dd-MS^2^, TopN = 10) with a mass resolution of 17,500. Fragmentation was performed using higher-energy collisional dissociation (HCD).

#### Grouping and model preparation

**Figure 1.**
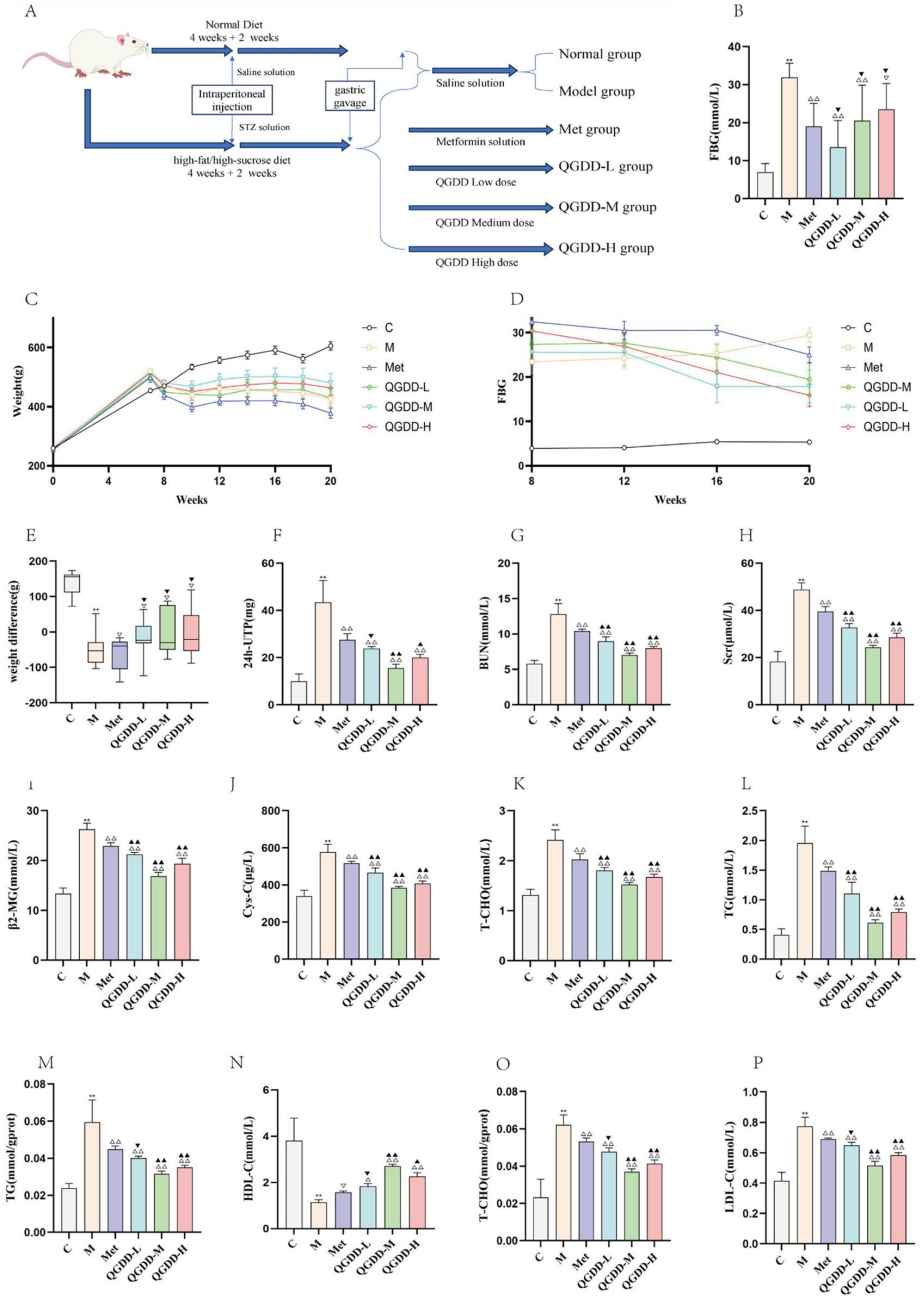
Experimental procedure, general condition, and biochemical indices of rats in each group. A: Flowchart of SD rat modeling and grouping; B: Bar charts of week 20 blood glucose levels across groups; C: Line chart showing dynamic changes in body weight of rats in each group from weeks 0 to 20; D: Line charts of blood glucose dynamics from weeks 8–20; E: Weight difference of each group; F: Bar chart of 24-UTP levels in rats from each group; G: Bar chart of serum BUN concentration in each group; H: Bar chart of serum scr concentration in each group; I: Bar chart of serum β2-MG concentration in each group; J: Bar chart of serum Cys-C concentration in each group; K: Bar graph depicting serum T-CHO concentrations in each group of rats; L: Bar graph depicting serum TG concentrations in each group of rats; M: Bar graph depicting renal tissue TG concentrations in each group of rats; N: Bar graph depicting renal tissue HDL-C concentrations in each group of rats; O: Bar graph depicting renal tissue T-CHO concentrations in each group of rats; P: Bar graph depicting renal tissue LDL-C concentrations in each group of rats; Compared to group C, **p < 0.01, *p < 0.05, ^ns^p > 0.05; Compared to group M, ^△△^*p* < 0.01, ^△^*p* < 0.05, ^▽^*p* > 0.05; Compared to the Met group, ^▲▲^*p* < 0.01, ^▲^*p* < 0.05, ^▼^*p* > 0.05.

After one week of adaptive feeding, SD rats were randomly assigned to six groups (*n* = 12 per group) by random number method: Blank Control Group, Model Control Group, Western Medicine Group, low-dose TCM group, medium-dose TCM group, and high-dose TCM group (hereinafter referred to as C Group, M Group, Met Group, QGDD-L Group, QGDD-M Group, and QGDD-H Group). 6 rats in a cage and 2 cages per group. Rats in the C Group were fed standard chow with ad libitum access to food and water, while the other five groups were fed a high-fat/high-sucrose diet (composition: 50% breeding rodent chow, 18.4% lard, 1.3% cholesterol, 0.3% bile salts, 15% sucrose, 12% casein, 2% premix, and 1% maltodextrin) with ad libitum access to food and water. After four weeks of feeding, body weights were recorded, followed by a 12-hour fasting period. After fasting, a freshly prepared STZ solution was intraperitoneally administered at 30 mg/kg based on body weight to rats in the M Group, the Met Group and the QGDD-L/M/H Groups. Blood glucose levels from tail veins were measured one and two weeks post-injection. Rats with random blood glucose readings exceeding 16.7 mmol/L in both measurements were considered successful DM models. After two additional weeks of feeding with the original diet, 24-hour urine was collected from the DM model rats. Rats exhibiting 24-hour urinary protein ≥30 mg were deemed successful DKD models. These rats continued on the original diet with ad libitum access to food and water until the experiment concluded. The QGDD-L/M/H groups were administered low, medium, and high doses *via* oral gavage at concentrations of 2.85 g/kg, 5.7 g/kg, and 8.5 g/kg, respectively. The C Group and M Group received equivalent volumes of normal saline *via* oral gavage. The treatment regimen continued for 144 consecutive days (Figure 1(A)). After that, all SD rats were fasted for 12 h with free access to water prior to anesthesia. Anesthesia was induced by intraperitoneal injection of 20% urethane solution at a dose of 1.2 g/kg *via* the abdominal aorta. After complete anesthesia was achieved, blood was collected from the abdominal aorta, and bilateral kidneys were excised. Following the completion of these procedures, the rats were euthanized by cervical dislocation.

#### H&E Staining

Renal tissue samples were collected and fixed in 4% paraformaldehyde solution for 24 h. The tissues were then processed through dehydration, embedding, and sectioning. Following this, the samples were cleared with xylene and dehydrated using a graded ethanol series. Eosin staining was then performed, and the sections were rinsed with double-distilled water, followed by hematoxylin staining. After washing again with double-distilled water, the specimens were mounted with neutral balsam. The samples were analyzed and observed under a microscope.

#### Masson staining

Kidney tissues fixed in 4% paraformaldehyde were processed, embedded in paraffin, and sectioned into 5 μm-thick slices. Masson’s trichrome staining was performed using a staining kit to examine fibrosis accumulation in kidney tissues. Finally, images were captured using a fluorescence microscope, followed by analysis of the fibrosis.

#### Transmission electron microscopy

Renal tissue blocks were fixed in pre-cooled 4% glutaraldehyde solution for 1 h, as glutaraldehyde possesses strong penetrability and effectively preserves fine ultrastructure. Following this, the tissues were further fixed in pre-cooled 1% osmium tetroxide for 2 h. The samples were then dehydrated through an ethanol series, followed by dehydration in 100% acetone. Infiltration was performed using a mixture of resin and acetone for 15 min, followed by pure resin infiltration for 30 min. Embedding was carried out using epoxy resin and a hardening agent. Ultrathin sections (approximately 40–50 nm thick) were prepared using an ultramicrotome, and selected sections were collected. Renal tissue sections underwent double staining: first with 3% uranyl acetate saturated in ethanol, followed by lead citrate staining. Finally, the ultrastructure of the renal tissues was observed under TEM and images were captured.

#### Oil Red O staining

Renal tissues fixed in 4% paraformaldehyde were processed, embedded in paraffin, and sectioned into 5 μm-thick slices. Modified Oil Red O staining was performed to assess lipid deposition in the renal tissues. Images were captured under a fluorescence microscope, followed by analysis of fibrosis.

#### Prussian blue staining

Renal tissues fixed in 4% paraformaldehyde were processed, embedded in paraffin, and sectioned into 5 μm-thick slices. Prussian blue staining was performed to evaluate iron deposition in the renal tissues. Images were captured under a fluorescence microscope, followed by analysis of fibrosis.

#### Detection by ELISA and kit-based methods

Whole blood samples were allowed to clot at room temperature for 30 min, followed by centrifugation at 1000 × g for 15 min to collect the supernatant. Serum levels of Cys-C and β2-MG in each group were measured using ELISA kits. Kit-based methods were employed to detect other relevant biomarkers. All experimental procedures were strictly conducted according to the manufacturer’s instructions.

#### Detection by TUNEL assay

Deparaffinized rat kidney tissue sections were rehydrated. After slight drying, sections were covered with Proteinase K working solution. Following three 5-minute PBS washes, sections were incubated with permeabilization working solution at room temperature for 20 min. After three additional PBS washes, sections were covered with buffer and incubated at room temperature for 10 min. Appropriate amounts of TdT enzyme, dUTP, and buffer from the TUNEL kit were mixed at a 1:5:50 ratio. The mixture was applied to cover the tissue sections, which were then placed flat in a humidity chamber and incubated at 37 °C for 2 h to maintain humidity. Sections were subsequently washed with PBS three times (5 min each). After PBS removal, DAPI staining solution was added dropwise and incubated at room temperature for 10 min, protected from light. Following three additional PBS washes (5 min each), sections were observed and imaged under a fluorescence microscope (DAPI UV excitation wavelength 330–380 nm, emission wavelength 420 nm, emitting blue fluorescence; CY3 excitation wavelength 510–561 nm, emission wavelength 590 nm, emitting red fluorescence). Under the microscope, blue fluorescence represents nuclei, while red fluorescence within the nuclei indicates apoptosis-positive cells. Mean optical density values were calculated using ImageJ software.

#### ROS detection

Rat kidney sections were fixed with 4% paraformaldehyde for 5 min, then rinsed with distilled water for 1 min. Sections were washed three times for 5 min each in PBS (pH 7.4) on a decoloring shaker. After slight drying, ROS staining solution (1 mM Dihydroethidium DMSO solution) was added dropwise, followed by incubation at 37 °C for 30 min in a light-protected incubator. The slides were placed in PBS (pH 7.4) and washed three times for 5 min each on a decolorizing shaker. After brief drying of the sections, DAPI staining solution was added within the encircled area and incubated for 10 min at room temperature, protected from light. The slides were washed three times in PBS (pH 7.4) for 5 min each on a decolorizing shaker. After brief drying, sections were mounted with anti-fade mounting medium. Observation and image capture were performed under a fluorescence microscope (DAPI ultraviolet excitation wavelength 330–380 nm, emission wavelength 420 nm; FITC green light excitation wavelength 465–495 nm, emission wavelength 515–555 nm; CY3 red light excitation wavelength 510–560 nm, emission wavelength 590 nm). Under the microscope, blue fluorescence represents nuclei, while red fluorescence within nuclei indicates ROS-positive expression. Mean optical density values were calculated using ImageJ software.

#### RT-qPCR detection

Total RNA was extracted from renal tissue of rats in each group and reverse transcribed into cDNA. Following the reagent kit instructions, GAPDH was used as the internal reference. mRNA levels of NRF2, HO-1, and GPX4 were analyzed using the 2^−ΔΔCt^ method. Primer sequences were synthesized by Fuzhou Shangya Biotechnology Co., Ltd., as listed in Table 1.

**Table 1.** Primer sequences for Nrf2, HO-1, GPX4, and GAPDH genes.

Gene	Forward primer	Reverse primer
Nrf2	CGATTAGAGGCTCATCTCACAA	GTTGAATTGCTCCTTGGACATC
HO-1	CTAAGACCGCCTTCCTGCTC	GCGGTGTCTGGGATGAACTA
GPX4	GGCAGGAGCCAGGAAGTAAT	TGGGCATCGTCCCCATTTAC
GAPDH	ACGGCAAGTTCAACGGCACAG	GAAGACGCCAGTAGACTCCACGAC

#### Western blot analysis

Total protein was extracted from rat renal tissues, and protein concentrations for each group were quantified using a BCA kit. An appropriate loading buffer was prepared, and proteins were denatured at high temperature before being stored at −20 °C for later use. Gels of suitable concentration were pre-prepared, and equal volumes of samples were loaded into gel wells for electrophoresis. The transfer buffer was pre-chilled. After electrophoresis, gels and PVDF membranes were assembled into transfer cassettes following a designated sequence. Protein transfer was performed at a constant current of 400 mA for an optimized duration. Membranes were then blocked with 5% skim milk/BSA (0.1 g/mL) and washed three times with TBST (10 min per wash). Primary antibodies against Nrf2, HO-1, and GPX4 were incubated separately, followed by three TBST washes. Corresponding secondary antibodies were incubated at room temperature, with subsequent washing of membranes three times in TBST. Protein bands were visualized using a chemiluminescent substrate and analyzed using ImageJ software.

#### Statistical processing

Data are expressed as mean ± standard deviation. The normality of data distribution was assessed using the Shapiro-Wilk test, and homogeneity of variances was evaluated with Levene’s test. For comparisons among multiple groups, one-way ANOVA followed by LSD post hoc test was applied when data met assumptions of normality and equal variance; otherwise, the Kruskal-Wallis test with Mann-Whitney U post hoc comparisons (Bonferroni-corrected) was used. All analyses were performed using SPSS 27.0, and a P-value < 0.05 was considered statistically significant.

### Randomization and blinding

Animals were randomly assigned to six groups (*n* = 12 per group) using a random number table. To minimize bias, investigators responsible for outcome assessments (including biochemical assays, histological evaluation, and data analysis) were blinded to the group allocation throughout the experiment.

## Results

### Component analysis of QGDD

In negative ion mode, 292 compounds were identified, among which linoleic acid exhibited the highest score, followed by 16-hydroxyhexadecanoic acid (Table 2). In positive ion mode, 210 compounds were detected, with chrysin demonstrating the highest score, succeeded by kaempferol (Table 3).

**Table 2.** Information on the top 20 compounds with the highest MS2 score in negative ion mode.

ID	MS2 name	MS2 ppm	MS2 score	RT [min]	Formula	Molecular Weight
1718	Linoleic acid	−0.91	100	12.841	C18 H32 O2	280.2402
1592	16-Hydroxyhexadecanoic acid	−0.09	99.8	9.903	C16 H32 O3	272.2351
1541	Genistein	−0.17	99.8	6.659	C15 H10 O5	270.0528
1766	Stearic acid	−0.63	99.7	10.775	C18 H36 O2	284.2715
141	4-Oxoproline	−9.98	99.6	0.917	C5 H7 N O3	129.0426
1375	Palmitoleic acid	−0.92	99.6	12.583	C16 H30 O2	254.2246
1930	(+/-)9-HODE	−0.59	99.5	10.443	C18 H32 O3	296.2351
142	4-Oxoproline	−9.98	99.5	1.173	C5 H7 N O3	129.0426
157	Methylsuccinic acid	−8.73	99.5	2.658	C5 H8 O4	132.0423
2187	(±)12(13)-DiHOME	−0.19	99.4	8.981	C18 H34 O4	314.2457
2186	(±)9(10)-DiHOME	−0.19	99.4	9.09	C18 H34 O4	315.2457
1737	Oleic acid	−1.22	99.4	13.561	C18 H34 O2	282.2559
192	Salicylic acid	−9.2	99.3	3.755	C7 H6 O3	138.0317
660	Citric acid	−4.59	99.2	0.88	C6 H8 O7	192.027
1362	Daidzein	−1.43	99.2	5.319	C15 H10 O4	254.0579
1057	Myristic acid	−2.45	99.2	12.34	C14 H28 O2	228.2089
297	2-Hydroxybenzothiazole	−7.28	99.1	5.815	C7 H5 N O S	151.0092
661	Citric acid	−4.61	99.1	1.172	C6 H8 O7	192.027
856	Jasmonic acid	−2.84	99.1	5.763	C12 H18 O3	210.1256
437	2-Mercaptobenzothiazole	−5.93	99	6.596	C7 H5 N S2	166.9863

**Table 3.** Information on the top 20 compounds with the highest MS2 score in positive ion mode.

ID	MS2 name	MS2 ppm	MS2 score	RT [min]	Formula	Molecular Weight
2070	Chrysin	−2.7	99.8	8.1	C15 H10 O4	254.0572
2376	Kaempferol	−2.21	99.8	6.747	C15 H10 O6	286.0471
2221	Genistein	−3.09	99.7	6.677	C15 H10 O5	270.052
534	Maltol	−0.06	99.6	3.224	C6 H6 O3	126.0317
1137	2-Mercaptobenzothiazole	−1.71	99.4	6.608	C7 H5 N S2	166.9861
2412	Catechin	−1.83	99.4	3.296	C15 H14 O6	290.0785
2222	Emodin	−3.09	99.4	6.524	C15 H10 O5	270.052
1305	2-(Methylthio)benzothiazole	−2.05	99.3	8.72	C8 H7 N S2	181.0016
419	Indole	0.63	99.3	3.126	C8 H7 N	117.0579
793	6-Methylquinoline	−1.16	99.2	3.695	C10 H9 N	143.0733
390	D-(+)-Proline	1.49	99.1	0.697	C5 H9 N O2	115.0635
692	Trigonelline	−1.51	99.1	0.796	C7 H7 N O2	137.0475
2219	Emodin	−3.09	99	5.285	C15 H10 O5	270.052
921	Guanine	−1.14	98.9	1.396	C5 H5 N5 O	151.0492
922	Guanine	−0.92	98.9	1.136	C5 H5 N5 O	151.0493
2188	Adenosine	−1.7	98.8	1.509	C10 H13 N5 O4	267.0963
3351	Astragaloside A	−4.85	98.6	6.947	C41 H68 O14	784.4571
2367	Biochanin A	−2.17	98.6	11.039	C16 H12 O5	284.0679
391	D-(+)-Proline	1.59	98.6	0.801	C5 H9 N O2	115.0635

### Renal biochemistry

#### General conditions and body weight

Baseline body weights were similar across all rat groups at week 0. Throughout the experiment, rats in the C group displayed good general health, with active behavior, intact and glossy fur, normal food and water intake, and regular urination and defecation. Body weight increased steadily over time. In contrast, rats in the M group exhibited lethargy, reduced mobility, hunched posture, rough, dull fur with hair loss, polydipsia, polyphagia, loose stools, and increased urine output, along with progressive weight loss (Figure 1(C,E)). At week 20, body weight in the M Group was significantly reduced compared to the C group (*p* < 0.01). Rats in the QGDD-L, QGDD-M, and QGDD-H groups showed increased body weight at week 20, but the changes were not statistically significant (*p* > 0.05). The Met group also experienced a decrease in body weight at week 20, but the difference was not statistically significant (*p* > 0.05). Compared to the Met group, the QGDD-M/H groups exhibited statistically significant weight gain at week 20 (*p* < 0.01), while the QGDD-L group showed a weight increase without statistical significance (*p* > 0.05) (Table 4). Overall, the Met group and QGDD-L/M/H groups demonstrated varying degrees of improvement compared to the M Group.

**Table 4.** Body weight of rats in each group of weeks 0 and week 20.

Gruop	n	Week 0	Week 20
C	8	259.1 ± 9.8	591.3 ± 41.2
M	8	257.8 ± 8.3 ^ns^	451.7 ± 38.7**
Met	8	255.3 ± 14.0 ^ns▽▼^	420.1 ± 48.9^▽^
QGDD-L	8	256.0 ± 9.5 ^ns▽▼^	457.0 ± 77.4^▽▼^
QGDD-M	8	252.9 ± 10.2 ^ns▼▽^	502.7 ± 79.3^▽▲▲^
QGDD-H	8	260.7 ± 6.6 ^ns▼▽^	480.1 ± 59.1^▽▲▲^

#### Fasting blood glucose results

Analysis across different time points revealed that FBG concentrations in the C Group rats remained relatively stable. The M Group showed a rapid initial increase in FBG levels, followed by a gradual rise. Both the Met group and the QGDD-L/M/H groups exhibited a gradual decrease in FBG concentration, with the most pronounced reduction observed in the QGDD-M group (Figure 1(D)). At corresponding time points, compared to the C Group, FBG levels in the M Group rats were significantly elevated, with a statistically significant difference (*p* < 0.01), confirming the successful establishment of the DKD model. In comparison to the M Group, FBG concentrations significantly decreased in the Met group and in the QGDD-L/M/H groups at both the 8th and 12th weeks of intervention (*p* < 0.01), with the most notable reduction in the QGDD-M group. After 12 weeks of intervention, FBG concentrations in the QGDD-L/M/H groups showed more significant decreases compared to the Met group, with statistically significant differences (*p* < 0.05) (Figure 1(B) and Table 5).

**Table 5.** Fasting blood glucose of rats in each group of week 0, 8, 12, 16, 20.

Group	n	0	8	12	16	20
C	8	4.1 ± 0.5	3.9 ± 0.5	4.1 ± 0.7	5.4 ± 1.1	5.3 ± 0.6
M	8	5.0 ± 1.5 ^ns^	23.5 ± 8.3	24.2 ± 7.0	25.3 ± 6.4	29.5 ± 5.1**
Met	8	5.4 ± 0.3 ^ns▽^	32.5 ± 2.5	30.5 ± 6.1	30.5 ± 3.3	25.0 ± 5.2^△△^
QGDD-L	8	5.7 ± 1.1 ^ns▽▼^	25.6 ± 8.0	25.5 ± 8.2	17.9 ± 10.4	17.9 ± 10.4^△△▼^
QGDD-M	8	5.3 ± 0.5 ^ns▽▼^	27.3 ± 9.2	27.7 ± 6.0	24.4 ± 8.4	19.5 ± 10.2^△△▼^
QGDD-H	8	5.4 ± 1.6 ^ns▽▼^	30.4 ± 8.0	26.9 ± 8.3	21.0 ± 10.7	15.9 ± 8.0^△▼^

For all Tables above, Compared to Group C, **p < 0.01, *p < 0.05, ^ns^p > 0.05; Compared to Group M, ^△△^*p* < 0.01, ^△^*p* < 0.05, ^▽^*p* > 0.05; Compared to the Met group, ^▲▲^*p* < 0.01, ^▲^*p* < 0.05, ^▼^*p* > 0.05.

#### Results of urinary 24-UTP, serum BUN, Scr, Cys-C, and β2-MG levels

Urine and serum test results across groups revealed that, compared to the C Group, the M Group exhibited significantly elevated 24-hour urinary total protein (24-UTP) levels and increased serum BUN, Scr, Cys-C, and β2-MG levels (*p* < 0.01), confirming the successful establishment of the DKD model in rats. In contrast, the Met group and the QGDD-L/M/H groups showed significant reductions in 24-UTP and serum BUN, Scr, Cys-C, and β2-MG levels following intervention (*p* < 0.01). The QGDD-L group demonstrated statistically significant decreases in serum BUN, Scr, Cys-C, and β2-MG levels compared to the Met group (*p* < 0.01), though the reduction in 24-UTP was not statistically significant (*p* > 0.05). The QGDD-M group showed significant reductions in both 24-UTP and serum levels of BUN, Scr, Cys-C, and β2-MG (*p* < 0.01). The QGDD-H group exhibited significant reductions in 24-UTP and serum BUN (*p* < 0.05), Scr (*p* < 0.01), Cys-C (*p* < 0.01), and β2-MG (*p* < 0.01) (Figure 1(F-J)). These results suggest that QGDD, at all tested concentrations, improves serum levels of BUN, Scr, Cys-C, and β2-MG in DKD model rats, thereby enhancing renal function to varying degrees.

#### Results of lipid metabolism in renal tissue

Biochemical analysis of renal tissues and blood samples from rats in each group revealed the following findings: Compared to the C Group, the M Group exhibited significantly increased serum levels of triglycerides (TG), total cholesterol (TCHO), and low-density lipoprotein cholesterol (LDL-C), along with a decreased level of high-density lipoprotein cholesterol (HDL-C) (*p* < 0.01). Additionally, renal tissue TG and TCHO levels were significantly elevated in the M Group (*p* < 0.01). In contrast, the Met group and the QGDD-L/M/H groups showed significantly reduced serum TG, TCHO, and LDL-C levels (*p* < 0.01). Although HDL-C levels increased, the difference was not statistically significant (*p* > 0.05). Renal tissue TG and TCHO levels were also significantly decreased (*p* < 0.01). Compared to the Met group, the QGDD-L group showed significantly elevated serum TG, TCHO, and LDL-C levels (*p* < 0.01) and a statistically significant reduction in HDL-C (*p* < 0.05). Renal tissue TG and TCHO levels increased, but this was not statistically significant (*p* > 0.05). The QGDD-M group exhibited increased serum TG, TCHO, and LDL-C levels, accompanied by decreased HDL-C levels, while renal tissue TG and TCHO levels were significantly elevated (*p* < 0.01). Similarly, the QGDD-H group showed increased serum TG, TCHO, and LDL-C levels, decreased HDL-C levels, and significantly elevated renal tissue TG and TCHO levels (*p* < 0.01) (Figure 1(K-P)). These results suggest that QGDD at varying concentrations differentially improves lipid metabolism in the serum and renal tissues of DKD model rats, thus inhibiting renal lipid deposition.

#### Gross examination of rat kidneys in each group

**Figure 2.**
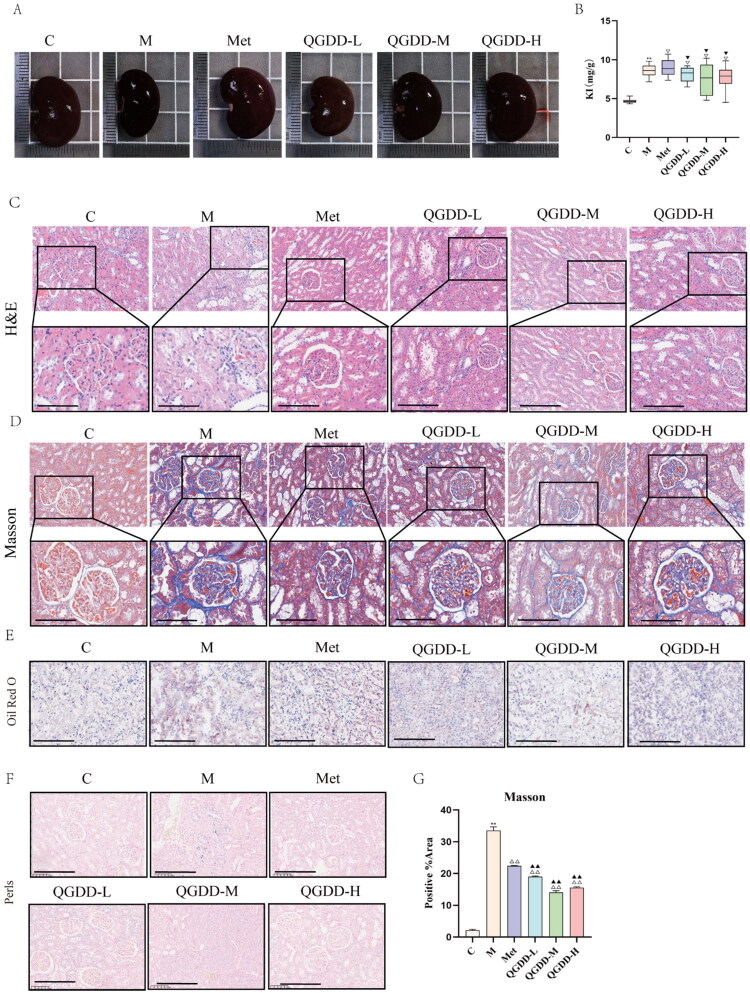
Results of gross kidney morphology and pathological sections in rats from each group. A: Gross morphology of rat kidneys from each group; B: Kidney index of rats from each group; C: HE staining of renal tissues from rats in each group (×200); D: Masson staining results of renal tissue from each group (×200); E: Oil Red O staining of renal tissues in each group (×200); F: Prussian blue staining of renal tissues in each group (×200); G: Bar charts of positive fibrosis rates in renal tissue from each rat group; Compared to group C, **p < 0.01, *p < 0.05, ^ns^p > 0.05; Compared to group M, ^△△^*p* < 0.01, ^△^*p* < 0.05, ^▽^*p* > 0.05; Compared to the Met group, ^▲▲^*p* < 0.01, ^▲^*p* < 0.05, ^▼^*p* > 0.05.

Compared with the C group, the M group exhibited increased kidney volume and a higher kidney index (*p* < 0.01). Compared with the M group, the Met, QGDD-L, QGDD-M, and QGDD-H groups showed a slight reduction in kidney volume and a decrease in the kidney index, although these differences were not statistically significant (*p* > 0.05). Compared with the Met group, the QGDD-L, QGDD-M, and QGDD-H groups demonstrated no significant change in kidney volume, and the kidney index was slightly lower, but again without statistical significance (*p* > 0.05) (Figure 2(A,B)). These results indicate that the gross renal morphology of DKD rats improved following intervention with metformin or different concentrations of QGDD compared to the nonintervention group, although no significant differences were observed among the various drug intervention groups.

#### Results of HE staining and masson staining in renal tissue

HE staining of renal tissue revealed that the C Group exhibited well-preserved renal histoarchitecture, with glomeruli, renal tubules, and interstitial structures displaying regular cellular morphology and orderly arrangement, without significant abnormalities. In contrast, the M Group showed severe disruption of renal architecture, characterized by irregular cellular morphology, disordered cellular arrangement, significant glomerular atrophy, markedly dilated Bowman’s space, reduced renal interstitial vasculature with edema, variably thickened basement membranes (BMs), focal dilatation of renal tubules, and mesangial matrix expansion, accompanied by inflammatory cell infiltration in the surrounding areas. Compared to the Model Group, the Met group and the QGDD-L/M/H groups demonstrated varying degrees of improvement in renal pathological damage (Figure 2(C)).

Masson staining of renal tissues indicated that, compared to the C Group, the M Group exhibited significantly increased collagen fiber deposition in the renal tubulointerstitium and considerable extracellular matrix accumulation. When compared to the M Group, the Met group and QGDD-L/M/H groups showed varying degrees of improvement in renal pathological damage (Figure 2(D)). Quantitative analysis of Masson-stained collagen fibers indicated that the collagen fiber area fraction was significantly higher in the M group than in the C group (*p* < 0.01). Compared with the M group, the collagen fiber area fraction was significantly reduced in the Met group and the QGDD-L/M/H groups (*p* < 0.01). Furthermore, the QGDD-L/M/H groups demonstrated a significant decrease in collagen fiber area fraction relative to the Met group (*p* < 0.01) (Figure 2(G)). These findings suggest that different concentrations of QGDD exert certain anti-renal fibrotic effects.

### Oxidative stress and ferroptosis

#### Results of Oil Red O and prussian blue staining in renal tissues

Oil Red O staining of renal tissues revealed no lipid deposition in the kidneys of the C Group. In contrast, the M Group exhibited noticeable lipid deposition, primarily localized within renal tubular cells. Compared to the Model Group, the Metformin group and the low-, medium-, and high-dose QGDD groups showed varying degrees of reduction in renal lipid deposition (Figure 2(E)).

Prussian blue staining demonstrated no iron deposition in the kidneys of the Blank Control Group, whereas blue iron deposits were observed in the renal tissues of the Model Group, scattered within renal tubular cells. Relative to the Model Group, the Metformin group and the low-, medium-, and high-dose QGDD groups exhibited varying degrees of reduction in renal iron deposition (Figure 2(F)).

#### Results of Fe^2+^, GSH, SOD, and MDA levels in renal tissue

**Figure 3.**
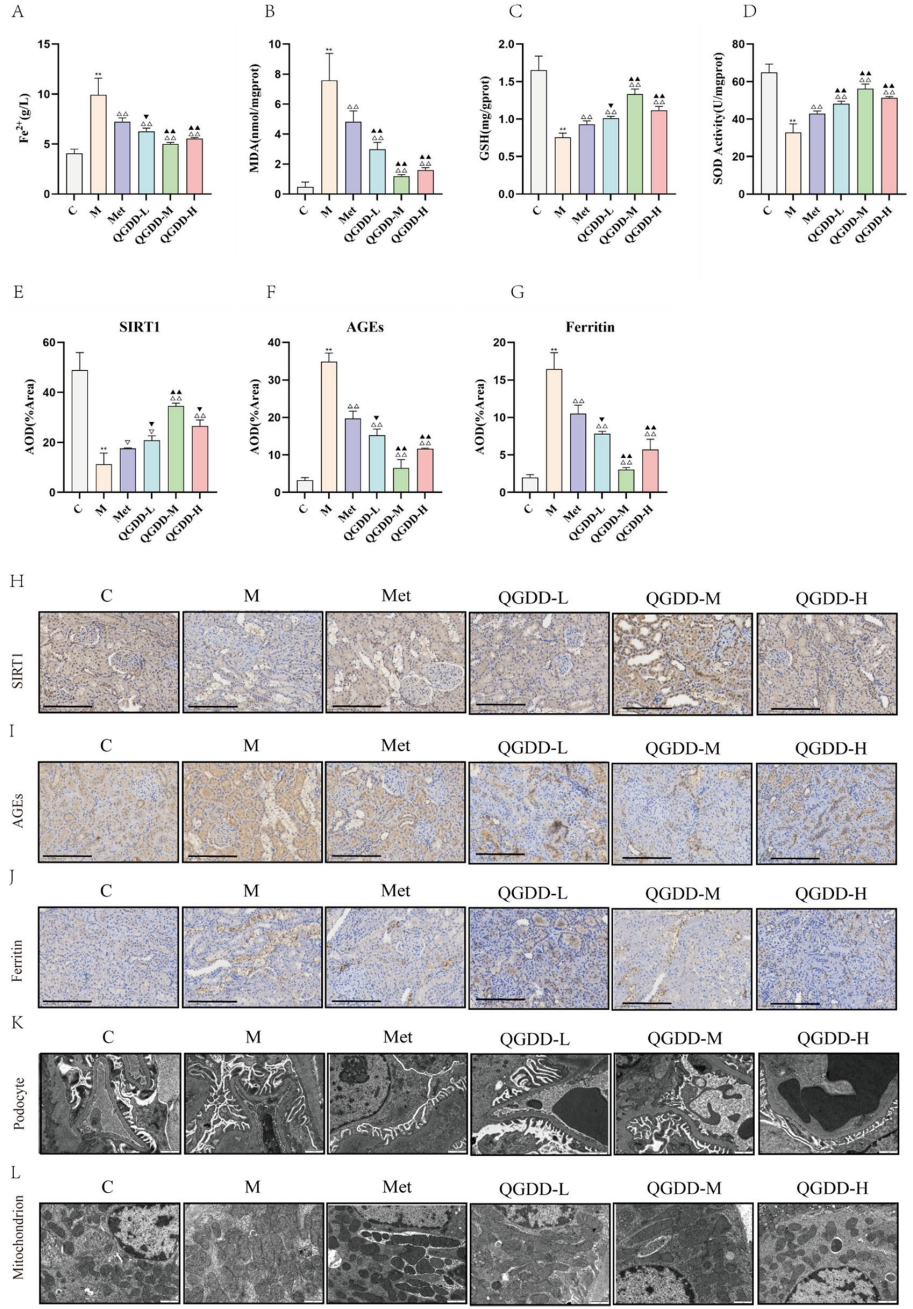
Results of ferroptosis markers, immunohistochemistry, and transmission electron microscopy in each group. A: Renal tissue Fe^2+^ content in each group of rats; B: Renal tissue malondialdehyde (MDA) content in each group; C: Renal tissue superoxide dismutase (SOD) activity in each group; D: Renal tissue glutathione (GSH) activity in each group; D: Bar charts of AOD of SIRT1 protein in renal tissue from each rat group; E: Bar charts of AOD of AGEs protein in renal tissue from each rat group; F: Bar charts of AOD of ferritin protein in renal tissue from each rat group; G-I: IHC staining results of renal tissue from each group; J-K: Transmission electron microscopy Images of renal podocytes and mitochondria (x2000); Compared to group C, **p < 0.01, *p < 0.05, ^ns^p > 0.05; Compared to group M, ^△△^*p* < 0.01, ^△^*p* < 0.05, ^▽^*p* > 0.05; Compared to the Met group, ^▲▲^*p* < 0.01, ^▲^*p* < 0.05, ^▼^*p* > 0.05.

Biochemical testing of renal tissue across groups revealed that, compared to the C Group, the M Group exhibited significantly increased Fe^2+^ and MDA content, along with decreased GSH and SOD activity (*p* < 0.01). In comparison to the M Group, both the Met group and the QGDD-L/M/H groups showed decreased Fe^2+^ and MDA content (*p* < 0.01), as well as increased GSH and SOD activity (*p* < 0.01) in renal tissue following intervention. Compared to the Met group, the QGDD-L group demonstrated decreased MDA content and increased SOD activity in renal tissue, with statistically significant differences (*p* < 0.05). Fe^2+^ content decreased, and GSH content and activity increased, though without statistically significant differences (*p* > 0.05). The QGDD-M group showed a significant reduction in Fe^2+^ and MDA content while increasing GSH content and SOD activity in renal tissue (*p* < 0.01). Similarly, the QGDD-H group significantly decreased Fe^2+^ and MDA content while enhancing GSH content and SOD activity in renal tissue (*p* < 0.01) (Figure 3(A–D)). These results suggest that QGDD, at all tested concentrations, reduces Fe^2+^ and MDA levels, while enhancing SOD and GSH activity in DKD model rats, thereby ameliorating oxidative stress and inhibiting ferroptosis.

#### Immunohistochemical results of SIRT1, AGEs, and ferritin in renal tissue

Immunohistochemical staining revealed significantly higher SIRT1 protein expression in the interstitial areas of renal tubules and blood vessels in the C group, with sparse expression of AGEs and Ferritin protein (Figure 3(H–J)). In contrast, the M Group exhibited markedly reduced SIRT1 expression, alongside significantly increased expression of AGEs and Ferritin (*p* < 0.01). Compared to the M Group, the QGDD-M and QGDD-H groups showed increased SIRT1 protein expression and decreased AGEs and Ferritin expression, with statistically significant differences (*p* < 0.01). The Met group and the QGDD-L group also demonstrated increased SIRT1 protein expression, though without statistical significance (*p* > 0.05), while both groups exhibited decreased AGEs and Ferritin expression with statistically significant differences (*p* < 0.01). Compared to the Met group, the QGDD-L group showed increased SIRT1 protein expression and decreased AGEs and Ferritin expression, though without statistically significant differences (*p* > 0.05). The QGDD-M group exhibited significantly increased SIRT1 protein expression and decreased AGEs and Ferritin expression (*p* < 0.01). In the QGDD-H group, SIRT1 expression increased without statistical significance (*p* > 0.05), while AGEs and Ferritin expression decreased with statistically significant differences (*p* < 0.01) (Figure 3(E–G)). These results indicate that QGDD, at different concentrations, can activate SIRT1 expression, thereby inhibiting ferroptosis and ameliorating oxidative stress in renal tissue.

#### TEM findings of renal tissues

TEM examination of cellular ultrastructure and mitochondria revealed the following observations: In the C Group, renal tissue cells displayed regular morphology, with well-defined glomerular structures and orderly arranged cells. The BM exhibited smooth and uniform thickness, without significant thickening. Podocytes and foot processes were structurally intact and well-organized, and mitochondrial cristae remained preserved. In the M Group, the BM showed irregular thickening, accompanied by mesangial expansion and increased mesangial matrix. Podocytes and foot processes exhibited varying degrees of diffuse fusion or even disappearance, while mitochondrial cristae were disrupted, and mitochondria appeared shortened. Compared to the M Group, both the Met group and the groups treated with different concentrations of QGDD displayed varying degrees of alleviation in renal pathological damage (Figure 3(K–L)). These findings suggest that different concentrations of QGDD exert protective effects on renal ultrastructure (e.g., podocytes) and mitochondrial morphology in renal tissue cells.

#### TUNEL assay results of renal tissue

**Figure 4.**
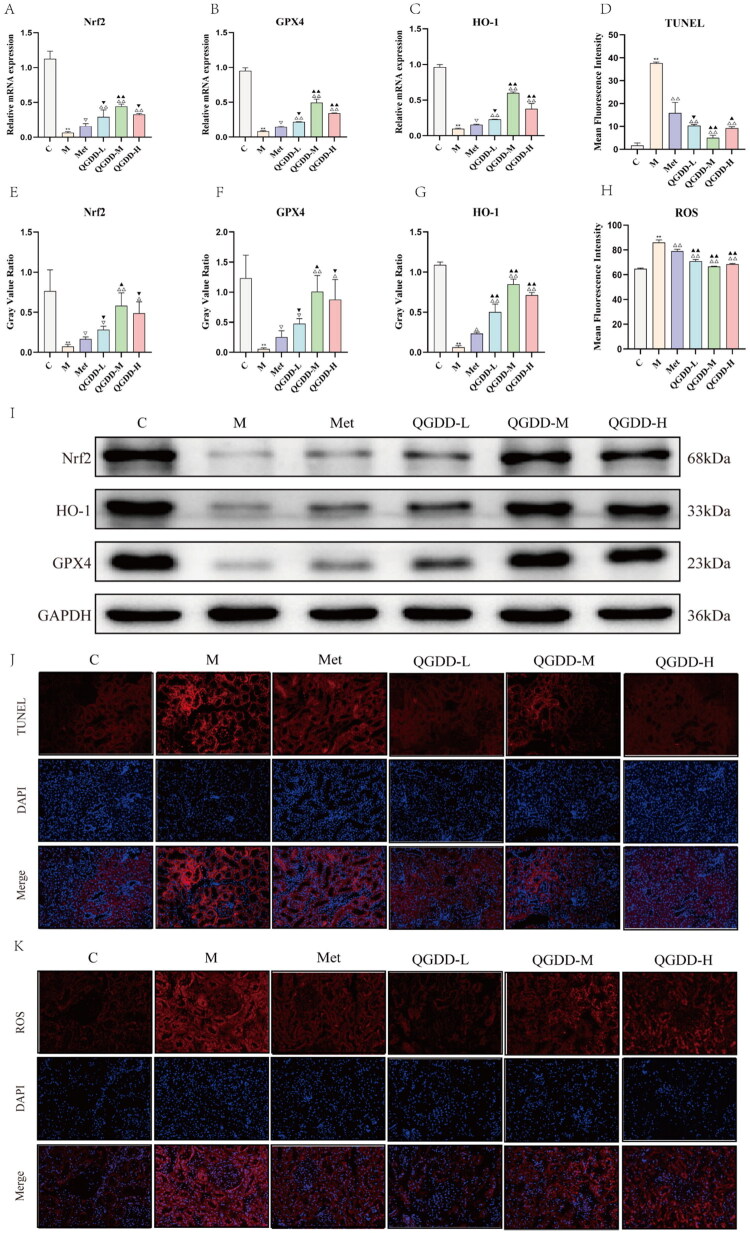
Results of mRNA and protein expression, as well as ROS and TUNEL fluorescence assays in each group. A: Bar chart of Nrf2 qPCR expression intensity in renal tissue across experimental groups; B: Bar chart showing GPX4 qPCR expression levels in renal tissue across experimental groups; C: Bar chart of HO-1 qPCR expression intensity in renal tissue across experimental groups; D: Bar chart showing mean fluorescence intensity of TUNEL in rats from each group; E: Bar chart of Nrf2 protein expression levels in renal tissues across groups; F: Bar chart of GPX4 protein expression levels in renal tissues of rats across groups; G: Bar chart of HO-1 protein expression levels in renal tissues of rats across groups; H: Bar chart showing mean ROS fluorescence intensity in renal tissue of rats across groups; I: Western blot protein bands of Nrf2, GPX4, HO-1, and GAPDH in renal tissues of rats across groups; J: TUNEL fluorescence-stained sections of renal tissue from rats in each group (x200); K: ROS fluorescence staining of renal tissue sections (×200) in rats from each group; Compared to group C, **p < 0.01, *p < 0.05, ^ns^p > 0.05; Compared to group M, ^△△^*p* < 0.01, ^△^*p* < 0.05, ^▽^*p* > 0.05; Compared to the Met group, ^▲▲^*p* < 0.01, ^▲^*p* < 0.05, ^▼^*p* > 0.05.

Frozen section analysis of renal tissue revealed higher fluorescence signals from TUNEL staining in the M Group compared to the C Group, indicating increased apoptosis in renal cells of DKD model rats. In comparison to the M Group, the Met group and the QGDD-L/M/H groups exhibited lower TUNEL fluorescence signals in renal tissue, suggesting varying degrees of apoptosis reduction in rats from both the Met group and the QGDD-L/M/H groups (Figure 4(D)). Quantitative analysis revealed that compared with Group C, the fluorescence signal was enhanced in Group M (*p* < 0.01). In comparison with Group M, the fluorescence signals were attenuated in the Met group and the QGDD-L/M/H groups (*p* < 0.01). Furthermore, relative to the Met group, the fluorescence signals in the QGDD-L/M/H groups were also reduced (*p* > 0.05, *p* < 0.01, *p* < 0.05) (Figure 4(J)).

#### ROS results in renal tissue

Frozen section analysis of renal tissue revealed that, compared to the C Group, the M Group exhibited stronger ROS fluorescence signals, indicating higher ROS accumulation in the kidneys of DKD model rats. This confirms the successful establishment of the DKD model as intended in this study. In comparison to the M Group, renal tissue from rats in the Met group and the QGDD-L/M/H groups showed lower ROS fluorescence signals, indicating varying degrees of attenuation in the inflammatory response (Figure 4(H)). Quantitative analysis revealed that compared with Group C, the fluorescence signal was enhanced in Group M (*p* < 0.01). In comparison with Group M, the fluorescence signals were attenuated in the Met group and the QGDD-L/M/H groups (*p* < 0.01). Furthermore, relative to the Met group, the fluorescence signals in the QGDD-L/M/H groups were also reduced (*p* < 0.01) (Figure 4(K)).

#### RT-qPCR results of Nrf2, GPX4, and HO-1 in renal tissue

Real-time qPCR results demonstrated that, compared to the C Group, mRNA expression levels of Nrf2, HO-1, and GPX4 in the renal tissue of rats in the M Group were significantly downregulated, with statistically significant differences (*p* < 0.01). In comparison to the M Group, the Met group showed upregulated mRNA expression levels of Nrf2, GPX4, and HO-1 in renal tissue, but without statistical significance (*p* > 0.05). The QGDD-L/M/H groups exhibited upregulated mRNA expression levels of Nrf2, GPX4, and HO-1 in renal tissue, with statistically significant differences (*p* < 0.01). Compared to the Met group, the mRNA expression levels of Nrf2, GPX4, and HO-1 in renal tissue were upregulated in the QGDD-L group, but without statistical significance (*p* > 0.05). The QGDD-M group showed statistically significant upregulation of Nrf2, GPX4, and HO-1 mRNA expression in renal tissue (*p* < 0.01). In the QGDD-H group, mRNA expression of GPX4 and HO-1 in renal tissue was significantly upregulated (*p* < 0.01), while Nrf2 expression showed an increase without statistical significance (*p* > 0.05) (Figure 4(A–C)). These results indicate that QGDD, at varying concentrations, upregulated mRNA expression levels of Nrf2, GPX4, and HO-1 in DKD model rats to different extents, thereby alleviating oxidative stress in renal tissues and inhibiting ferroptosis.

#### Western blot analysis of Nrf2, GPX4, and HO-1 protein expression in renal tissue

Western blot results of renal tissue showed that, compared to the C Group, the M Group exhibited significantly reduced protein expression levels of Nrf2, GPX4, and HO-1 (*p* < 0.01). In comparison to the M Group, the Met group demonstrated upregulated HO-1 protein expression with statistical significance (*p* < 0.05), while GPX4 and Nrf2 protein expression levels increased, but these changes lacked statistical significance (*p* > 0.05). In the renal tissue of rats from the QGDD-L group, HO-1 protein expression levels showed a significant upregulation (*p* < 0.05), although GPX4 and Nrf2 protein expression levels exhibited an upward trend without statistical significance (*p* > 0.05). In the QGDD-M group, the expression levels of GPX4, HO-1, and Nrf2 proteins were all significantly upregulated, with statistically significant differences (*p* < 0.01). In the QGDD-H group, HO-1 protein expression levels were significantly upregulated (*p* < 0.01), while GPX4 and Nrf2 protein expression levels showed an increasing trend, though no statistically significant difference was observed (*p* > 0.05). Compared to the Met group, the QGDD-L group exhibited significantly upregulated HO-1 protein expression levels in renal tissue (*p* < 0.01), while GPX4 and Nrf2 protein expression levels increased, but the differences were not statistically significant (*p* > 0.05). The QGDD-M group showed significantly upregulated expression levels of GPX4 (*p* < 0.05), HO-1 (*p* < 0.01), and Nrf2 (*p* < 0.05). The QGDD-H group exhibited significantly upregulated HO-1 protein expression (*p* < 0.01), while GPX4 and Nrf2 protein expression levels increased without statistical significance (*p* > 0.05) (Figure 4(E–G,I)). These results suggest that Metformin and all doses of QGDD can upregulate the expression levels of Nrf2, GPX4, and HO-1 proteins in renal tissue of DKD model rats to varying degrees, thereby inhibiting ferroptosis and oxidative stress, thus protecting renal tissue.

## Discussion

The present study demonstrates that Qigui Didang Decoction (QGDD) exerts significant renoprotective effects in a DKD rat model exhibiting features of metabolic memory. The key findings can be summarized as follows: 1) QGDD improved glucolipid metabolism and renal function, showing efficacy superior to metformin in several aspects; 2) QGDD reduced renal lipid and Fe^2+^ deposition, alleviated oxidative stress, and suppressed ferroptosis; 3) Mechanistically, QGDD significantly upregulated the renal expression of SIRT1 and Nrf2, thereby enhancing downstream HO-1 and GPX4 levels. These results collectively suggest that the renoprotective effect of QGDD in a setting mimicking metabolic memory is associated with the activation of the SIRT1/Nrf2 signaling pathway and the inhibition of ferroptosis-related lipid peroxidation, providing an experimental basis for its multi-target therapeutic potential against sustained DKD pathology.

DKD is one of the most severe microvascular complications of diabetes mellitus, clinically characterized by persistently increased urinary protein excretion and progressive decline in glomerular filtration rate. Pathologically, DKD is marked by diffuse thickening of the glomerular BM and excessive matrix deposition in the mesangial area. These structural changes disrupt the function of the glomerular filtration barrier, leading to massive proteinuria. Research indicates that high-glucose environments activate oxidative stress, promote abnormal collagen deposition, accelerate BM thickening, and expand the mesangial matrix, which ultimately results in glomerulosclerosis (Zhang et al. 2022). Pathologically, DKD is marked by diffuse thickening of the glomerular basement membrane and mesangial matrix expansion, which are key drivers of glomerulosclerosis and proteinuria (Scilletta et al. 2023). In the present study, the DKD model rats exhibited these characteristic changes, which were ameliorated by QGDD treatment.

Ferroptosis, characterized by iron-dependent lipid peroxidation, has been implicated in DKD progression. Prior studies have shown that specific agents (e.g., puerarin, curcumin) can inhibit ferroptosis in various disease models by activating the SIRT1/Nrf2 pathway (Ji et al. 2023; Jiang et al. 2025). Extending these findings to the context of DKD and herbal formulations, our study provides direct evidence that the full prescription of QGDD can similarly upregulate the SIRT1/Nrf2/GPX4 axis in renal tissue. We observed that the DKD model exhibited a significant reduction in SIRT1 expression alongside decreased Nrf2, HO-1, and GPX4 levels, concordant with heightened oxidative stress and ferroptosis markers. QGDD intervention, particularly at the medium dose, effectively reversed these alterations, with an effect surpassing that of metformin. This positions the SIRT1/Nrf2 pathway as a plausible central hub through which QGDD remodels redox homeostasis and inhibits ferroptosis in the diabetic kidney.

As an NAD+-dependent histone deacetylase, SIRT1 plays a pivotal protective role in mitigating oxidative stress and metabolic disorders by regulating the expression of downstream antioxidant genes. Existing research indicates that SIRT1 activation inhibits ferroptosis by enhancing Nrf2 stability and promoting its nuclear translocation, thereby upregulating the expression of antioxidant proteins such as HO-1 and GPX4(Han et al. 2025; Yang et al. 2025; Zhang et al. 2025). Puerarin significantly reduces lipid peroxidation levels in cardiomyocytes of high-fat diet-fed rats and suppresses the accumulation of ferroptosis-related markers, MDA and Fe^2+^, by activating the SIRT1/Nrf2 pathway (Jiang et al. 2025). Additionally, reports indicate that melatonin effectively alleviates α-synuclein-induced neuronal ferroptosis in Parkinson’s disease models through the SIRT1/Nrf2/HO-1/GPX4 pathway (Lv et al. 2024). While this effect is pronounced in the central nervous system, our study demonstrates that, in the Model Group, SIRT1 protein expression was significantly reduced in renal tissue, accompanied by decreased mRNA and protein levels of Nrf2, HO-1, and GPX4. This finding aligns with the pathological characteristics of exacerbated oxidative stress and ferroptosis activation during the progression of DKD. Following intervention with varying concentrations of QGDD, SIRT1 expression was significantly upregulated, and the activity of Nrf2 and its downstream target genes was restored. This effect surpassed that of Metformin, suggesting that QGDD may inhibit ferroptosis in DKD kidneys by regulating the SIRT1/Nrf2 axis to remodel redox homeostasis. The SIRT1/Nrf2 pathway serves as a central hub for antioxidant defense and a critical link between ferroptosis and metabolic memory.

The interaction between SIRT1 and AGEs is particularly critical in DKD. The accumulation of AGEs accelerates renal fibrosis and exacerbates oxidative damage (Huang et al. 2024; Zhang et al. 2025). This study demonstrated that AGEs and ferritin expression in renal tissue from the Model Group were significantly elevated. However, both levels decreased markedly after intervention with various concentrations of QGDD, showing superior efficacy compared to Metformin. Notably, these reductions were negatively correlated with SIRT1 expression. This observation aligns with research by Zhuang et al. who found that Formononetin reduced AGEs deposition and renal tubule damage in DKD models by activating the SIRT1/Nrf2 pathway (Zhuang et al. 2020).

The multi-component nature of QGDD likely underlies its regulatory effect on the SIRT1/Nrf2 pathway. Individual herbs within QGDD, such as *Astragalus membranaceus* (containing astragaloside IV) and *Angelica sinensis* (containing ferulic acid), have been reported to modulate SIRT1 or Nrf2 activity in other disease contexts (Tang et al. 2022; Liu et al. 2025). Our study advances this knowledge by demonstrating that the complete QGDD prescription can effectively activate this pathway and inhibit ferroptosis in a holistic DKD model. This observation aligns with the TCM theory of “multiple components, multiple targets,” suggesting that the integrated effect of the full formula may differ from and potentially surpass the simple sum of its individual parts, offering a unique advantage in modulating complex diseases like DKD.

Although previous studies have suggested that individual herbs or active components in QGDD may influence the SIRT1/Nrf2 pathway (Wang et al. 2023; Sun et al. 2024), this study is the first to systematically demonstrate in a comprehensive animal model of DKD that the full prescription of QGDD can synergistically regulate this pathway and inhibit renal ferroptosis, thereby ameliorating metabolic memory. This highlights the advantage of traditional Chinese medicine (TCM) compound prescriptions in their holistic regulation *via* “multiple components-multiple targets,” an effect that may differ from the simple additive effects of single components.

In this study, metformin was selected as the positive control because it is a foundational therapeutic agent for type 2 diabetes and provides effective glycemic control. The results showed that, under comparable glycemic control, medium- and high-dose QGDD demonstrated superior effects compared to metformin in improving renal function indicators, alleviating renal oxidative stress and ferroptosis, and upregulating the expression of key proteins in the SIRT1/Nrf2 pathway. This suggests that the renoprotective effect of QGDD may be partially independent of its glucose-lowering effect and is associated with its unique mechanism of modulating the SIRT1/Nrf2/ferroptosis axis.

A notable finding was that the medium dose (5.7 g/kg) of QGDD consistently yielded optimal efficacy, outperforming the higher dose. This non-linear dose-response relationship may be inherent to complex herbal formulations. One plausible explanation is the “saturation effect”; the medium dose may sufficiently activate the core SIRT1/Nrf2 antioxidant pathway, beyond which additional dosage provides no linear benefit. Furthermore, at higher doses, the dynamic interactions among the numerous active constituents within QGDD may change, potentially leading to antagonistic effects or the manifestation of minor side effects from certain components, thereby offsetting the overall therapeutic gain. This finding underscores the existence of an “optimal therapeutic window” for QGDD and highlights the critical importance of dose optimization in translating herbal medicine from preclinical research to precise clinical application.

Simultaneously, as a multi-component herbal formula, its non-linear dose-response relationship may also stem from dynamic changes in the interactions among different active constituents at higher concentrations. At elevated doses, the effects of certain components (e.g., active ingredients from herbs promoting blood circulation and resolving stasis) may become more pronounced, leading to more complex antagonistic or counteracting effects with other protective constituents within the formula.

This finding carries clear translational significance: it suggests the existence of an “optimal therapeutic window” for QGDD efficacy in clinical application, indicating that a higher dose is not necessarily better. This provides important experimental evidence for the precise, individualized dose adjustment—”tailoring treatment to the individual”—in the traditional Chinese medicine management of diabetic kidney disease (DKD).

### Strengths and limitations

This study possesses several strengths, including a multi-level evaluation from physiological phenotypes to molecular mechanisms, and the identification of an optimal medium dose, which provides crucial pre-clinical evidence for future clinical dose optimization.

This study found that the renal protective effect of QGDD is associated with the inhibition of ferroptosis through the regulation of the SIRT1/Nrf2/GPX4 axis. This pathway has been confirmed to be equally critical in human DKD (Zhou et al. 2025), and the proven efficacy of similar natural products (Wang et al. 2022; Liang et al. 2025) collectively supports its translational potential. Future clinical studies, based on clarifying the pharmacokinetics of QGDD, are required to validate its value in the multi-target synergistic treatment of DKD.

However, several limitations should be acknowledged. First, while our data strongly associate QGDD’s benefits with SIRT1/Nrf2 activation and ferroptosis inhibition, the *in vivo* correlation design precludes definitive causal inference. Future studies employing genetic knockdown (e.g., SIRT1 siRNA) or pharmacological inhibitors of this pathway are needed to establish mechanistic causality. Second, the findings are derived from a single-sex (male) rat model of DKD, necessitating caution in extrapolation to females and human patients. Third, as a multi-component formula, the precise active compounds responsible for the observed effects warrant identification through further serum pharmacochemistry and formula deconstruction studies. Finally, although our model exhibits persistent injury reminiscent of metabolic memory, we lack direct evidence that QGDD reverses underlying epigenetic alterations. Future investigations measuring specific histone modifications or DNA methylation patterns associated with metabolic memory would be valuable to address this gap.

## Conclusions

In conclusion, this study demonstrates that Qigui Didang Decoction alleviates renal injury in a DKD model with metabolic memory features. The renoprotective effects are associated with the activation of the SIRT1/Nrf2 signaling pathway and the inhibition of ferroptosis. Notably, the medium dose (5.7 g/kg) showed optimal efficacy, underscoring the importance of dose optimization. These findings provide novel experimental support for QGDD as a multi-target therapeutic candidate for DKD.

## Data Availability

The data that support the findings of this study are available from the corresponding author, [Min Lin], upon reasonable request.

## References

[CIT0001] Alicic RZ, Rooney MT, Tuttle KR. 2017. Diabetic kidney disease challenges, progress, and possibilities. Clin J Am Soc Nephrol. 12(12):2032–2045. 10.2215/CJN.11491116 28522654 PMC5718284

[CIT0002] Chen Y et al. 2025. Ellagic acid protects against acute stress-induced kidney damage in rats by regulating the SIRT1/nrf2 signaling pathway. J. Food Biochem. 2025(1):34631331. 10.1155/jfbc/3463133

[CIT0003] Dong Y, Zhang K, Chu J, Chu Q. 2025. [Didang decoction-medicated serum enhances autophagy in high glucose-induced rat glomerular endothelial cells via the PI3k/akt/mTOR signaling pathway]. Nan Fang Yi Ke Da Xue Xue Bao. 45(3):461–469. 10.12122/j.issn.1673-4254.2025.03.03 40159960 PMC11955890

[CIT0004] Fan L et al. 2024. Mechanism of qigui didang decoction in regulating metabolic memory of diabetic kidney disease based on GEO chip data mining and network pharmacology. Pharmacol Clin Chin Materia Medica. 40(07):20–26. 10.13412/j.cnki.zyyl.20240221.001

[CIT0005] Guo Y et al. 2024. Mechanism of qigui didang decoction in treatment of diabetic kidney disease based on network pharmacology and experimental verification. Moderniz Traditional Chinese Medicine Materia Medica-World Sci Technol. 26(10):2648–2661. 10.11842/wst.20230505009

[CIT0006] Han Q, Gu Y, Qian Y. 2025. Study on the mechanism of activating SIRT1/nrf2/p62 pathway to mediate autophagy-dependent ferroptosis to promote healing of diabetic foot ulcers. Naunyn Schmiedebergs Arch Pharmacol. 398(3):3015–3025. 10.1007/s00210-024-03400-4 39320410

[CIT0007] He J et al. 2022. Ferroptosis and ferritinophagy in diabetes complications. Mol Metab. 60:101470. 10.1016/j.molmet.2022.101470 35304332 PMC8980341

[CIT0008] Huang L et al. 2024. Injectable, anti-collapse, adhesive, plastic and bioactive bone graft substitute promotes bone regeneration by moderating oxidative stress in osteoporotic bone defect. Acta Biomater. 180:82–103. 10.1016/j.actbio.2024.04.016 38621599

[CIT0009] Jakubiak GK, Chwalba A, Basek A, Cieślar G, Pawlas N. 2024. Glycated hemoglobin and cardiovascular disease in patients without diabetes. J Clin Med. 14(1):53. 10.3390/jcm14010053 39797136 PMC11721913

[CIT0010] Jakubiak GK et al. 2025. Analysis of the relationship between glycated hemoglobin and echocardiographic parameters in patients without diabetes: a retrospective study. J Clin Med. 15(1):33. 10.3390/jcm15010033 41517283 PMC12786401

[CIT0011] Ji J et al. 2023. Emodin attenuates diabetic kidney disease by inhibiting ferroptosis via upregulating nrf2 expression. Aging (Albany NY). 15(15):7673–7688. 10.18632/aging.204933 37552124 PMC10457067

[CIT0012] Jiang S et al. 2025. Puerarin reduces susceptibility to ventricular arrhythmias and inhibits ferroptosis via sirt1/nrf2 signaling in high-fat-diet rats. Free Radic Biol Med. 227:472–484. 10.1016/j.freeradbiomed.2024.12.005 39647799

[CIT0013] Keating ST, El-Osta A. 2013. Glycemic memories and the epigenetic component of diabetic nephropathy. Curr Diab Rep. 13(4):574–581. 10.1007/s11892-013-0383-y 23639991

[CIT0014] Kushwaha K, Sharma S, Gupta J. 2020. Metabolic memory and diabetic nephropathy: beneficial effects of natural epigenetic modifiers. Biochimie. 170:140–151. 10.1016/j.biochi.2020.01.007 31954720

[CIT0015] Li X et al. 2022. Epigenetics in the pathogenesis of diabetic nephropathy. Acta Biochim Biophys Sin (Shanghai). 54(2):163–172. 10.3724/abbs.2021016 35130617 PMC9909329

[CIT0016] Liang Z et al. 2025. FGF21-engineered ADSCs promote diabetic wound healing by mitigating ferroptosis and oxidative stress via the SIRT1/NRF2/GPX4 signaling pathway. Stem Cell Res Ther. 16(1):650. 10.1186/s13287-025-04758-9 41257858 PMC12628967

[CIT0017] Liu P, Zhang Z, Chen Q. 2024. [Roles of ferroptosis in the development of diabetic nephropathy]. Zhejiang Da Xue Xue Bao Yi Xue Ban. 53(6):708–714. 10.3724/zdxbyxb-2024-0114 39757741 PMC11736350

[CIT0018] Liu Y et al. 2025. Pantothenic acid alleviates osteoarthritis progression by inhibiting inflammatory response and ferroptosis through the SIRT1/nrf2 signaling pathway. Chem Biol Interact. 413:111494. 10.1016/j.cbi.2025.111494 40157627

[CIT0019] Lu C, Jiang B, Xu J, Zhang X, Jiang N. 2023. Neferine protected cardiomyocytes against hypoxia/oxygenation injury through SIRT1/nrf2/HO-1 signaling. J Biochem Molecular Tox. 37(8):e23398. 10.1002/jbt.23398 37421224

[CIT0020] Lv X, Jiang J, An Y. 2024. Investigating the potential mechanisms of ferroptosis and autophagy in the pathogenesis of gestational diabetes. Cell Biochem Biophys. 82(1):279–290. 10.1007/s12013-023-01196-3 38214812

[CIT0021] Ren D, Li J, Chang B, Li C, Yang J. 2017. Early intervention with didang decoction delays macrovascular lesions in diabetic rats through regulating AMP-activated protein kinase signaling pathway. Chin J Nat Med. 15(11):847–854. 10.1016/S1875-5364(18)30018-9 29329611

[CIT0022] Scilletta S et al. 2023. Update on diabetic kidney disease (DKD): focus on non-albuminuric DKD and cardiovascular risk. Biomolecules. 13(5):752. 10.3390/biom13050752 37238622 PMC10216614

[CIT0023] Sun B et al. 2024. Quercetin inhibits ferroptosis through the SIRT1/nrf2/HO-1 signaling pathway and alleviates asthma disease. Transl Pediatr. 13(10):1747–1759. 10.21037/tp-24-193 39524399 PMC11543135

[CIT0024] Sun X et al. 2016. Activation of the p62-keap1-NRF2 pathway protects against ferroptosis in hepatocellular carcinoma cells. Hepatology. 63(1):173–184. 10.1002/hep.28251 26403645 PMC4688087

[CIT0025] Tang X, Li X, Zhang D, Han W. 2022. Astragaloside-IV alleviates high glucose-induced ferroptosis in retinal pigment epithelial cells by disrupting the expression of mir-138-5p/sirt1/nrf2. Bioengineered. 13(4):8240–8254. 10.1080/21655979.2022.2049471 35302431 PMC9162003

[CIT0026] Tong Q et al. 2024. Integrated chemical characterization, metabolite profiling, and pharmacokinetics analysis of zhijun tangshen decoction by UPLC-q/TOF-MS. Front Pharmacol. 15:1363678. 10.3389/fphar.2024.1363678 38523634 PMC10957775

[CIT0027] Villeneuve LM, Natarajan R. 2010. The role of epigenetics in the pathology of diabetic complications. Am J Physiol Renal Physiol. 299(1):F14–F25. 10.1152/ajprenal.00200.2010 20462972 PMC2904177

[CIT0028] Wang X et al. 2023. Ferulic acid activates SIRT1-mediated ferroptosis signaling pathway to improve cognition dysfunction in wilson’s disease. Neuropsychiatr Dis Treat. 19:2681–2696. 10.2147/NDT.S443278 38077239 PMC10710261

[CIT0029] Wang X et al. 2022. Astragaloside IV regulates the ferroptosis signaling pathway via the nrf2/SLC7a11/GPX4 axis to inhibit PM2.5-mediated lung injury in mice. Int Immunopharmacol. 112:109186. 10.1016/j.intimp.2022.109186 36115280

[CIT0030] Yang BSK et al. 2025. Update on strategies to reduce early brain injury after subarachnoid hemorrhage. Curr Neurol Neurosci Rep. 25(1):14. 10.1007/s11910-024-01396-1 PMC13081737 39722093

[CIT0031] Yuan C et al. 2025. S1r mediates NRF2 dependent ferroptosis of renal tubular epithelial cells to promote renal fibrosis in diabetic nephropathy. Int J Med Sci. 22(4):955–970. 10.7150/ijms.104324 39991769 PMC11843141

[CIT0032] Zhang J et al. 2022. Crocin protects the renal tubular epithelial cells against high glucose-induced injury and oxidative stress via regulation of the SIRT1/nrf2 pathway. Iran J Basic Med Sci. 25(2):193–197. 10.22038/IJBMS.2022.51597.11708 35655597 PMC9124533

[CIT0033] Zhang S et al. 2023. Carnosine alleviates kidney tubular epithelial injury by targeting NRF2 mediated ferroptosis in diabetic nephropathy. Amino Acids. 55(9):1141–1155. 10.1007/s00726-023-03301-5 37450047

[CIT0034] Zhang Y et al. 2025. Edaravone alleviates sepsis-induced diaphragmatic dysfunction via sirt1/nrf2 pathway. Int Immunopharmacol. 153:114475. 10.1016/j.intimp.2025.114475 40106902

[CIT0035] Zhang Y, Hu X, Chen S, Hua F, Zeng Z. 2025. Unveiling the impact of ferroptosis on diabetes-associated cognitive decline through comprehensive single-cell RNA sequencing and experimental studies. Febs J. 292(14):3795–3813. 10.1111/febs.70101 40254912

[CIT0036] Zhou Y et al. 2025. Activation of the lp-PLA2/LPC axis triggers endothelial ferroptosis to drive diabetic kidney disease. Free Radic Biol Med. 241:818–828. 10.1016/j.freeradbiomed.2025.09.036 40983197

[CIT0037] Zhuang K et al. 2020. Formononetin activates the nrf2/are signaling pathway via sirt1 to improve diabetic renal fibrosis. Front Pharmacol. 11:616378. 10.3389/fphar.2020.616378 33519483 PMC7845558

